# IgG Expression upon Oral Sensitization in Association with Maternal Exposure to Ovalbumin

**DOI:** 10.1371/journal.pone.0148251

**Published:** 2016-02-04

**Authors:** Rucheng Chen, Xiaoqiao Tang, Bolin Fan, Jiafa Liu, Xudong Jia, Xiaoguang Yang

**Affiliations:** 1 Hubei Provincial Key Laboratory for Applied Toxicology, Hubei Provincial Center for Disease Control and Prevention, Wuhan, Hubei, China, 430079; 2 College of Basic Medical Science, Zhejiang Chinese Medical School, Hangzhou, Zhejiang, China, 310053; 3 China National Center for Food Safety Risk Assessment, Beijing, China, 100021; 4 National Institute for Nutrition and Food Safety, Chinese Center for Disease Control and Prevention, Beijing, China, 100021; Mie University Graduate School of Medicine, JAPAN

## Abstract

The role of maternal allergen exposure in the allergenicity of the offspring remains controversial. Some studies have shown that maternal exposure is a risk factor for allergy in the offspring, whereas other studies have shown that maternal exposure induces immune tolerance and protects offspring from allergy disease. Therefore, we utilized maternal rat allergen exposure model to evaluate the offspring immune reactions to ovalbumin protein and to determine whether the Brown Norway (BN) rat model is a suitable animal model for studying the allergenicity of food proteins. For three generations, rats received an allergens or non-allergens by gavage during the pregnancy and lactation periods. After weaning, the offspring rats were used for oral sensitization experiment. In the sensitization experiment, the control rat, which had maternal exposure to phosphate-buffered saline (PBS), exhibited full response of IgG to oral exposure to OVA. The IgG level was significantly lower in F1 rats that were sensitized by maternal exposure to ovalbumin(OVA). Moreover, the lowest IgG level was found for the F3b sensitized by maternal rats exposed to OVA allergen for three continuous generations. Compared with maternal OVA exposure prior to postnatal sensitization, the sensitization via maternal PBS led to a higher serum level of OVA-specific IgG. However, the OVA-specific IgG levels for the two generations of maternal PBS exposure prior to postnatal sensitization was not higher than that for the one generation of maternal rats exposed to PBS prior to postnatal sensitization. Our studies demonstrate that maternal OVA exposure during the pregnancy and lactation can affect the results of oral sensitization studies using ovalbumin protein. BN rats must be bred in non-allergen conditions for at least one generation to avoid problems in rat models for studying the allergenicity of food proteins.

## Introduction

Food allergies are a food intolerance reaction mediated by immune processes. Type I(IgE-mediated) hypersensitivity reactions play a major role in food allergies. A series of adverse reaction in the human body, including death from anaphylactic shock, can be induced by food allergies[1].The incidence of food allergy has greatly increased over the past decade[2]; at present, the food allergy incidence in adults is estimated at 1–3%, whereas in young children, this rate is as high as 5–8%[3]. Food allergies are associated with adverse outcomes, and the rapidly increasing prevalence of allergic problems is a major global health issue. Food allergens are mostly proteins, although only a few dietary proteins can cause allergic reactions. Approximately 90% of these reactions come from eight types of food, that is, peanuts, soy, milk, eggs, fish, shellfish, wheat, and nuts. Other proteins, including the proteins in one hundred and sixty types of food can also induce allergic disease[4]. Additionally, new proteins that are produced by gene recombination have the potential to induce allergenic reactions and other adverse effects. Therefore, genetically modified foods have received significant attention in recent years.

For safety reasons, it is important to evaluate the allergenicity of protein-rich foods, including traditional and genetically modified foods. A decision tree strategy, as suggested by the International Life Sciences Institute(ILSI) Allergy and Immunology Institute and the International Food Biotechnology Council(IFBC) in recent years, represents the best-known allergy assessment protocol[5]. This strategy involves amino acid sequence comparisons, physical and chemical property studies, protein level considerations, and other approaches[5–7]. Such assessment methods may be associated with the potential risks of allergic reactions to expressed proteins; however, final conclusions regarding potential risks are not determined using these methods. Therefore, the joint Food and Agriculture Organization of the United Nations and the World Health Organization(FAO/WHO) consultation on biotechnology and food safety introduced serum screening and animal models into the decision tree strategy. Recently, animal models have been deemed a helpful tool for evaluating potential food allergens. Animal researchers have developed widely accepted animal models for food safety evaluations, including mice, guinea pigs, and rats. However, there are currently no well-validated animal models to evaluate food allergens.

In recent years, many studies have shown Brown Norway (BN) rats to be a suitable model for studying food allergens[8–10]. First, a high immunoglobulin (particularly IgE and IgG) response is induced in BN rats after ovalbumin gavage dosing. Moreover, after sensitization, the immune reactions to allergens in BN rats and humans are similar. Second, similar clinical reactions are observed in BN rats and humans after oral allergen challenge[11, 12], such as increased gut permeability as well as changes in blood pressure and respiratory function. Last, the most important advantage of the BN rat model over other animal models is that oral challenge can be conducted without an adjuvant, which mimics the route of exposure in humans[13, 14].

Our laboratory has been working to develop an oral sensitization BN rat model. We selected OVA, a well-defined chicken allergen, as a model allergen. Specifically, BN rats were dosed with OVA by gavage for 42 days. In our study, BN rats were successfully sensitized by a daily oral gavage dosing protocol. The allergen induced a high immunoglobulin (particularly IgG) response, elevated histamine levels and decreased blood pressure. Although the results from our study are similar to those of other studies[15–18], there were limitations in our early studies, as we did not consider pre-exposure and parental dietary effect factors in this model. In particular, the development of immune reactions induced by maternal allergen exposure remains controversial. Two prospective birth cohort studies (Isle of Wight, UK, and Avon Longitudinal Study of Parents and Children) reported no effect of maternal diets on the development of allergy[19–21]. In contrast, in other studies of infants that were at high risk for allergies, IgE and IgG sensitization to allergens in the infants was positively correlated with allergen consumption by their mothers during pregnancy[22, 23]. The widely accepted opinion is that exposure to an allergen from a parental generation may negatively affect oral sensitization immune reactions in the offspring. Indeed, some studies have shown that oral exposure to allergens may induce immunologic tolerance in rats. Therefore, at least two generations of animals should be bred on a diet free of the allergen under investigation before BN rats are used as an animal model[24].

Because of these uncertainties, the BN rat model may be greatly affected by maternal exposure. To address this issue, we investigated different immune reactions in offspring whose two parental generations of BN rats were gavage-dosed with an allergen or bred on a diet free of the allergen. The results of our study shed light on the influence of maternal allergen exposure on offspring immune reactions and help to determine the suitability of the BN rat as an animal model to study food protein allergens.

## Materials and Methods

### Animals

BN rats, 8–10 weeks old, were obtained from Vital River Laboratory Animal Technology Co. Ltd.(Beijing,China). The BN rats were housed in an animal room that was maintained at 24±1°C and 50±10% relative humidity with a 12-hlight/dark cycle. During the experiment and for at least 10 days prior to study initiation, the rats were fed a milk- and egg-free diet, excluding the allergen protein that was under investigation. Food and water were provided ad libitum. All of the animal studies were carried out in strict accordance with the recommendations of our institutional animal care and use committee. The protocol was approved by the committee on the ethics of animal experiments of Hubei Provincial Center for Disease Control and Prevention (Permit Number: 20140624004). All surgeries were performed under sodium pentobarbital anesthesia, and all efforts were made to minimize suffering.

### Allergens

OVA, a well-defined food allergen, was obtained from Sigma Chemicals, USA. Before the beginning of an experiment, 10mg/ML concentrated OVA solutions were made, divided into aliquots, and frozen at -20°C. The concentrated solutions were diluted to 1 mg/ml OVA solutions, which were used for daily dosing.

### Breeding protocols

The initial rats(F0) were dosed with 1 ml of the working 1 mg/mL OVA solution or phosphate-buffered saline(PBS) by gavage during pregnancy and lactation. The first generation(F1) rats were bred from PBS-exposed F0 rats, as control experiment rats(F0_matPBS_), and from OVA-exposed F0 rats as F1 experiment rats(F0_matOVA_).

In the second generation (F2), the F1 rats (F0_matOVA_) were dosed with 1 mL of 1 mg/mL OVA solution or PBS by gavage during pregnancy and lactation. The rats were bred from PBS-exposed F1 rats, which were denoted as F2a experiment rats(F0_matOVA_/F1_matPBS_), or from OVA-exposed F1 rats, which were denoted as F2b experiment rats(F0_matOVA_/F1_matOVA_).

**Fig 1:**
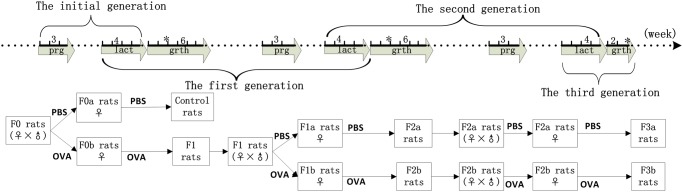
The three-generation breeding protocol. The solid line represents BN rats that were raised over the time line. The maker “prg” represents the pregnancy period. The maternal rats were dosed with PBS or OVA during the pregnancy period for 3 weeks. The maker “lact” represents the lactation period. The maternal rats were dosed with PBS or OVA during the lactation period for 3 weeks. “Grth” represents the growth period. The BN rats were raised on a standard diet during the growth period. For the 6 weeks following birth, the maternal rats were dosed with PBS or OVA during the lactation period, which lasted for 4 weeks. The offspring rats(control, F1, F2a, F2b, F3a, and F3b) were raised on a standard diet for the next 2 weeks. Some rats were chosen for sensitization studies from the control, F1, F2a, F2b, F3a, and F3b groups after 6 weeks; the others were persistently raised on routine diet for 4 weeks prior to mating.

In the third generation (F3), the F2a rats(F0_matOVA_/F1_matPBS_) were dosed with 1 ml PBS, and the F2b rats(F0_matOVA_/F1_matOVA_) were dosed with 1 ml of 1 mg/ml OVA solution daily by gavage during pregnancy and lactation. The F3a experimental rats(F0_matOVA_/F1_matPBS_/F2_matPBS_) were bred from the F2a rats, and the F3b experiment rats(F0_matOVA_/F1_matOVA_/F2_matOVA_) were bred from the F2b rats (Fig 1).

### Sensitization protocols

The rats used in the oral sensitization studies included the control, F1, F2a, F2b, F3a, and F3b rats after they were weaned. Each of the experiment rats was randomly divided into OVA and non-OVA groups, with 16 rats(eight male and eight female) in each group. The OVA and non-OVA groups were administered1 mL of 1 mg/mL OVA or 1 mL PBS daily by gavage, respectively, for 6 weeks. Blood samples were obtained at the 2^nd^, 4^th^, and 6^th^ weeks from the orbital plexus and centrifuged for 15 min at 2,000r/min at 4°C to obtain the sera. The sera were stored at −20°C until analyzing OVA-specific IgG levels with the enzyme-linked immunosorbent assay(ELISA) technique.

### OVA-specific IgG antibody assay

Antigen-specific IgG levels were determined using an ELISA technique. An OVA solution(10 μg/mL) was bound to 96-well microtiter plates(100 μL/well) overnight at 4°C. The plates were then washed three times with tap water containing 0.5% Tween 20. This step was followed by the addition of 100 μL/well PBS containing 0.5% Tween 20 and 2% sheep serum albumin. After a 1-h incubation at 37°C, the plates were washed, and 1:32 dilutions of rat serum were added to the wells and incubated for 1 h at 37°C. After washing, 100 μL/well peroxidase-conjugated goat anti-rat IgG(H+L) (sigma, USA) was added. After incubation for 1 h at 37°C, the plates were washed again, and an enzyme-substrate solution of tetramethylbenzidine(Sigma, 100 μL/well) was added. The plates were developed at room temperature for 10 min. Finally, 100 μL/well of 1 mol/L H_2_SO_4_ was added. Optical densities (OD values) were read at 492 nm with an ELISA plate reader. In all of the experiments, the non-OVA group that was dosed with PBS was used as the negative group, and the OVA group that was dosed with OVA was used as the positive group. A P/N value, which was a ratio of the positive and negative group OD values, was calculated for each experiment. A P/N value>2 was considered to indicate a positive serum response.

### Statistical analysis

Data are presented as P/N values, i.e., the ratios of the OVA group OD value divided by the non-OVA group OD value. A P/N IgG value >2 was considered to indicate positive expression. A non-parametric test(Mann-Whitney U) was used to analyze IgG expression in the offspring rats after maternal OVA exposure in the same generation of two experimental groups. One-way ANOVAs were used to analyze IgG expression in offspring rats after maternal OVA exposure over three generations in greater than two experimental group experiments. All of the statistical analyses were performed with the SPSS 17.0 software package. The IgG expression level was considered significantly different at P <0.05.

## Results

To determine whether maternal exposure affected the immune reaction in offspring, the maternal rats were exposed to antigen during pregnancy and lactation. Then, the offspring rats were gavage-sensitized with OVA for 6 weeks after weaning.

### The first-generation oral sensitization experiment

**Fig 2:**
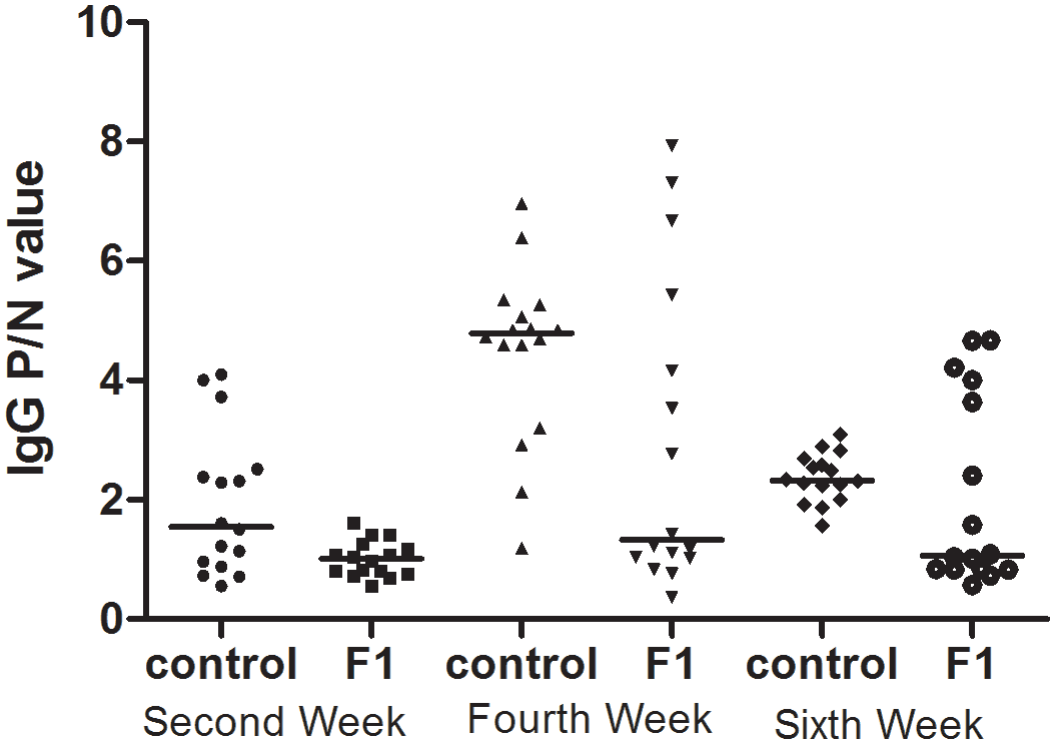
The serum IgG levels in first-generation rats. Maternal PBS exposure was used in the experimental control rats(F0_matPBS_), and maternal OVA exposure was used in the F1 experimental rats(F0_matOVA_), which were randomly divided into the OVA and non-OVA groups, respectively. The OVA group and non-OVA groups were administered treatments daily for 6 weeks, and blood samples were taken on the 2^nd^, 4^th^, and 6^th^ weeks from the orbital plexus. The number of animals per experiment was 32, and each group contained16 animals. The serum IgG levels were measured with an ELISA technique. Data are presented as P/N values representing the ratios of the OVA group OD values divided by the non-OVA group values in the respective experiment. P/N IgG values>2 were considered to indicate positive expression levels. The statistical analyses were performed with Non-parametric test(Mann-Whitney U), which compared the control and F1 rats. *p<0.05. **(Data in** S1 and S2 Tables)

In the first generations, the IgG-positive responses among maternal OVA-exposed rats(F1 rats) were different from those of maternal non-exposed rats(control rats).The positive rate in the F1 rats was 0(0/16), 43.8%(7/16), and 37.5%(6/16) at the 2^nd^, 4^th^, and 6^th^ weeks, respectively. We observed high IgG responses in the control rat group, in which 43.8%(7/16), 93.8%(15/16), and 81.3%(13/16) of animals responded at the 2^nd^, 4^th^, and 6^th^ weeks, respectively. Compared to the control rat group, maternal OVA exposure during pregnancy and lactation prior to postnatal sensitization clearly reduced the OVA-specific IgG levels. The IgG P/N values were decreased in the F1 rats at the 2^nd^ and 4^th^ weeks, and no differences in IgG value were detected between the F1 experiment and control experimental rats at the 6^th^ week(Fig 2).

### The second-generation oral sensitization experiment

**Fig 3:**
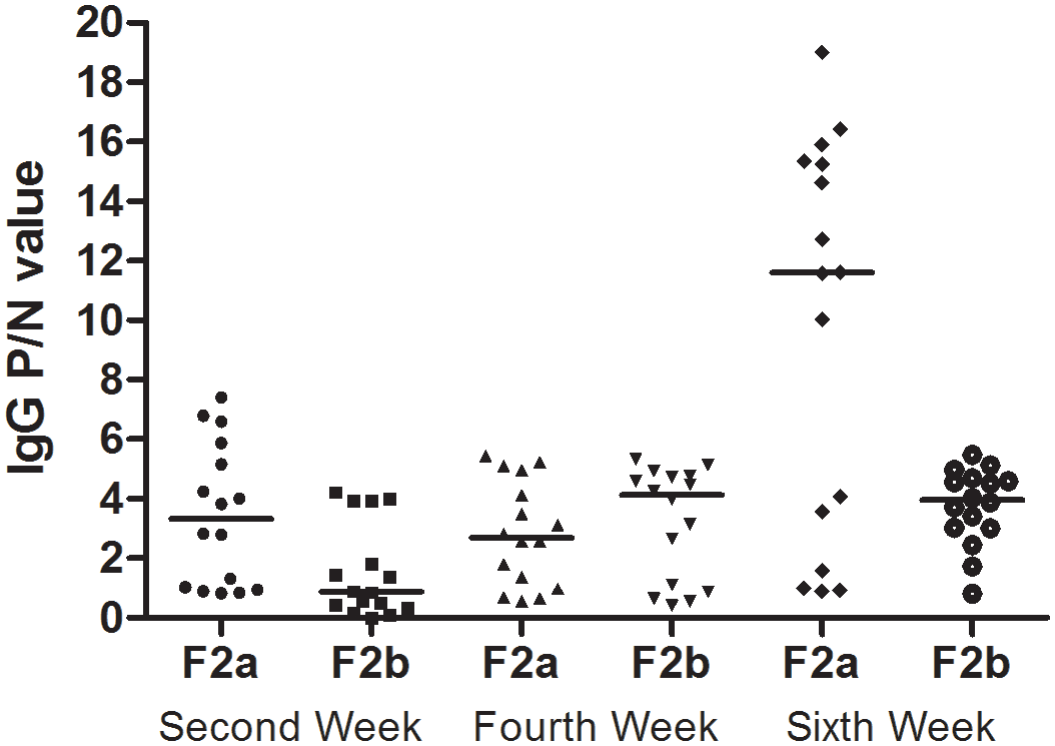
The serum IgG levels in second-generation rats. The F2a experiment rats, which included PBS-exposed F1 maternal rats(F0_matOVA/_F1_matPBS_), and F2b experiment rats, which included F1 maternal OVA-exposed rats(F0_matOVA_/F1_matOVA_), were randomly divided into OVA and non-OVA groups, respectively. The OVA group and non-OVA group were administered daily treatments daily for 6 weeks, and blood samples were taken on the 2^nd^, 4^th^, and 6^th^ weeks from the orbital plexus. The number of animals per experiment was 32, and each group contained16 animals. The serum IgG levels were measured with an ELISA technique. The data are presented as P/N values representing are the ratios of the OVA group OD values divided by the non-OVA group values in the respective experiment. P/N IgG values>2 were considered to indicate positive expression levels. The statistical analysis was performed with Non-parametric test(Mann-Whitney U) to compare the F2a and F2b experimental rats. *p<0.05. **(Data in** S3 and S4 Tables)

As shown in Fig 3, in the second generation, maternal OVA exposure led to lower IgG levels in the offspring, particularly at the 2^nd^ and 6^th^ weeks. The positive rates in the F2a(F0_OVAmat_/F1_PBSmat_) rats were 62.5%(10/16), 62.5%(10/16), and 75.0%(12/16) at the 2^nd^, 4^th^, and 6^th^ weeks, respectively. We observed positive IgG responses in the F2b(F0_OVAmat_/F1_OVAmat_) experimental rats, in which 25.0%(4/16), 68.8%(11/16), 87.5%(14/16) of animals responded at the 2^nd^, 4^th^, and 6^th^ weeks, respectively.

### The third-generation oral sensitization experiment

**Fig 4:**
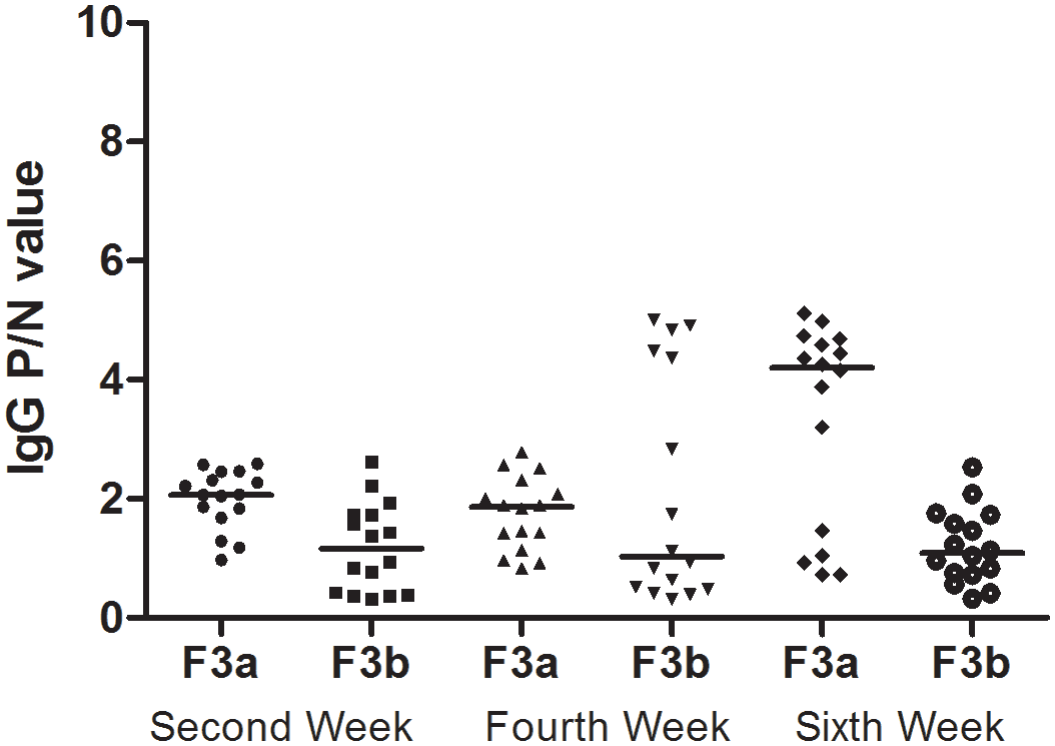
The serum IgG levels in third-generation rats. The F3a experiment rats, which included PBS-exposed F2a maternal rats (F0_matOVA_/F1_matPBS_/F2_matPBS_), and the F3b experiment rats, which included F2b maternal OVA-exposed rats(F0_matOVA_/F1_matOVA_/F2_matOVA_), were randomly divided into the OVA and non-OVA groups, respectively. The OVA and non-OVA groups were administered treatments daily for 6 weeks, and blood samples were taken on the 2^nd^, 4^th^, and 6^th^ weeks from the orbital plexus. The number of animals per experiment was 32, and each group contained16 animals. The serum IgG levels were measured with an ELISA technique. The data are presented as P/N values representing the ratios of the OVA group OD values divided by the non-OVA group values in the respective experiment. P/N IgG values>2 were considered to indicate positive expression levels. The statistical analysis was performed with Non-parametric test(Mann-Whitney U) to compare the F3a and F3b experimental rats. *p<0.05. **(Data in** S5 and S6 Tables)

As shown in Fig 4, in the third generation at the 2^nd^ and 6^th^ weeks, significant IgG P/N value differences between the F3a(F0_OVAmat_/F1_PBSmat_/F2_PBSmat_) and F3b(F0_OVAmat_/F1_OVAmat_/_F2OVAmat_) rats were observed. The IgG P/N values were significantly higher in the F3a rats than in the F3b rats. The positive rates in the F3a rats were 62.5%(10/16), 37.5%(6/16), and 68.8%(11/16) at the 2^nd^, 4^th^, and 6^th^ weeks, respectively. The positive IgG response rates in the F3b rat experiment were 12.5% (2/16), 37.5% (6/16), and 12.5% (2/16) at the 2^nd^, 4^th^, and 6^th^ weeks, respectively.

### Postnatal sensitization after maternal OVA exposure

**Fig 5:**
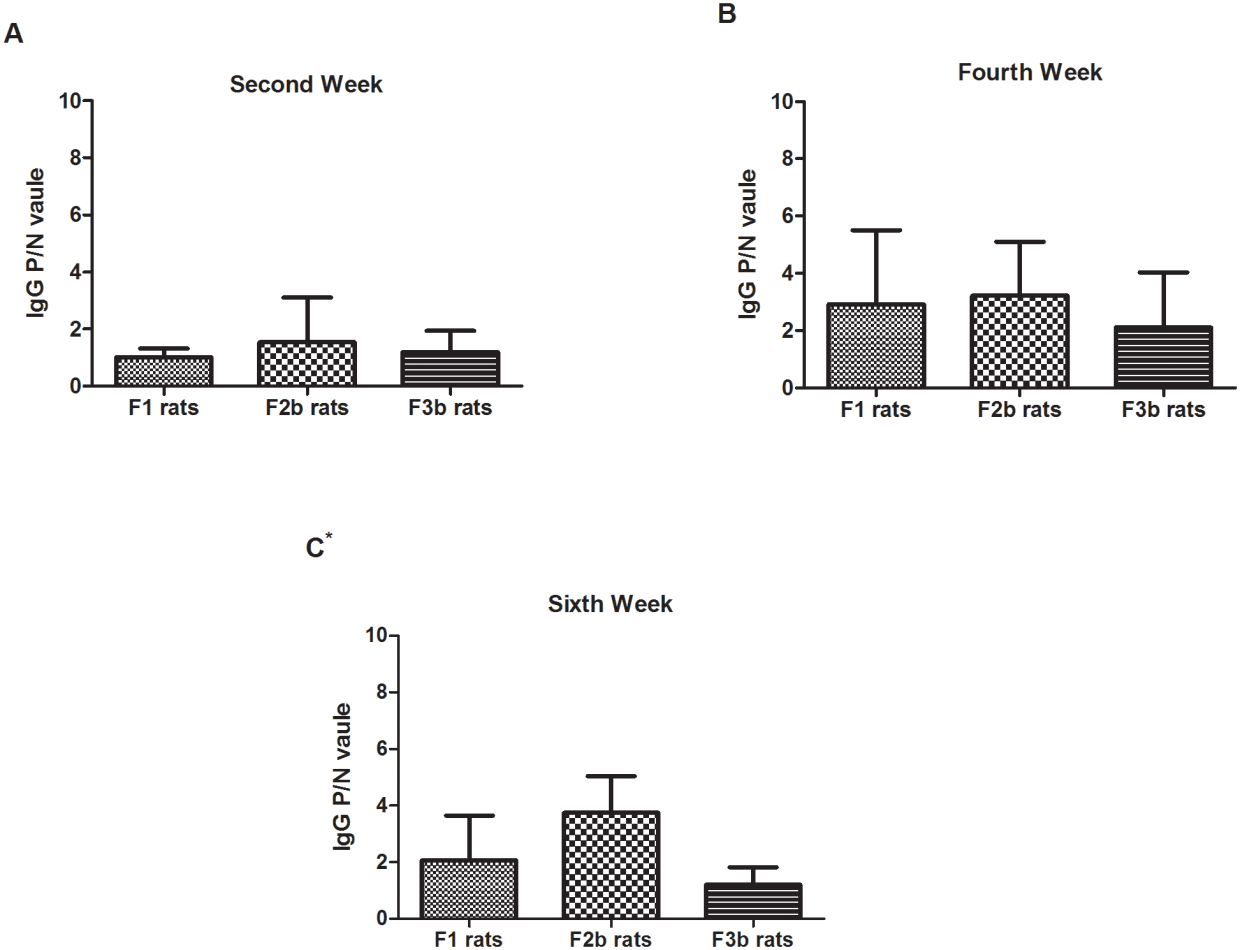
The serum IgG levels in the three generations after maternal OVA exposure. The F0 maternal OVA-exposedF1 experimental rats(F0_matOVA_), the F1 maternal OVA-exposed F2b experimental rats(F0_matOVA_/F1_matOVA_) and the F2b maternal OVA-exposed F3b experimental rats(F0_matOVA_/F1_matOVA_/F2_matOVA_) were randomly divided into OVA and non-OVA groups. The OVA and non-OVA groups were administered treatments daily for 6 weeks, and blood samples were taken on the 2^nd^(A), 4^th^(B), and 6^th^(C) weeks from the orbital plexus. The number of animals per experiment was 32, and each group contained 16 animals. The serum IgG levels were measured with an ELISA technique. The data are presented as P/N values representing the ratios of the OVA group OD values divided by the non-OVA group values in the respective experiment. P/N IgG values>2 were considered to indicate positive expression levels. The statistical analysis was performed with one-way ANOVA comparisons of the control and experimental F1 rats. *p<0.05. **(Data in** S2, S4 and S6 Tables)

The maternal rats were gavage-dosed with a 1 mg/mL OVA solution during pregnancy and lactation for three generations. OVA-specific IgG was measured at the 2^nd^, 4^th^ and 6^th^ weeks in the offspring rats(F1, F2b, and F3b). As indicated in Fig 5, the OVA-specific IgG response was low in the F1, F2b and F3b rat sera during the sensitization periods, especially at the 2^nd^ week. Despite the low OVA-specific IgG response, the level was still higher in the F1, F2b and F3b rats at the end of the sensitization period. Additionally, compared with the first- or second-generation OVA-exposed rats, the F3a rats, which had three generations of parental rats with continuous OVA exposure, generated the lowest OVA-specific IgG levels.

### Postnatal sensitization after maternal PBS exposure

**Fig 6:**
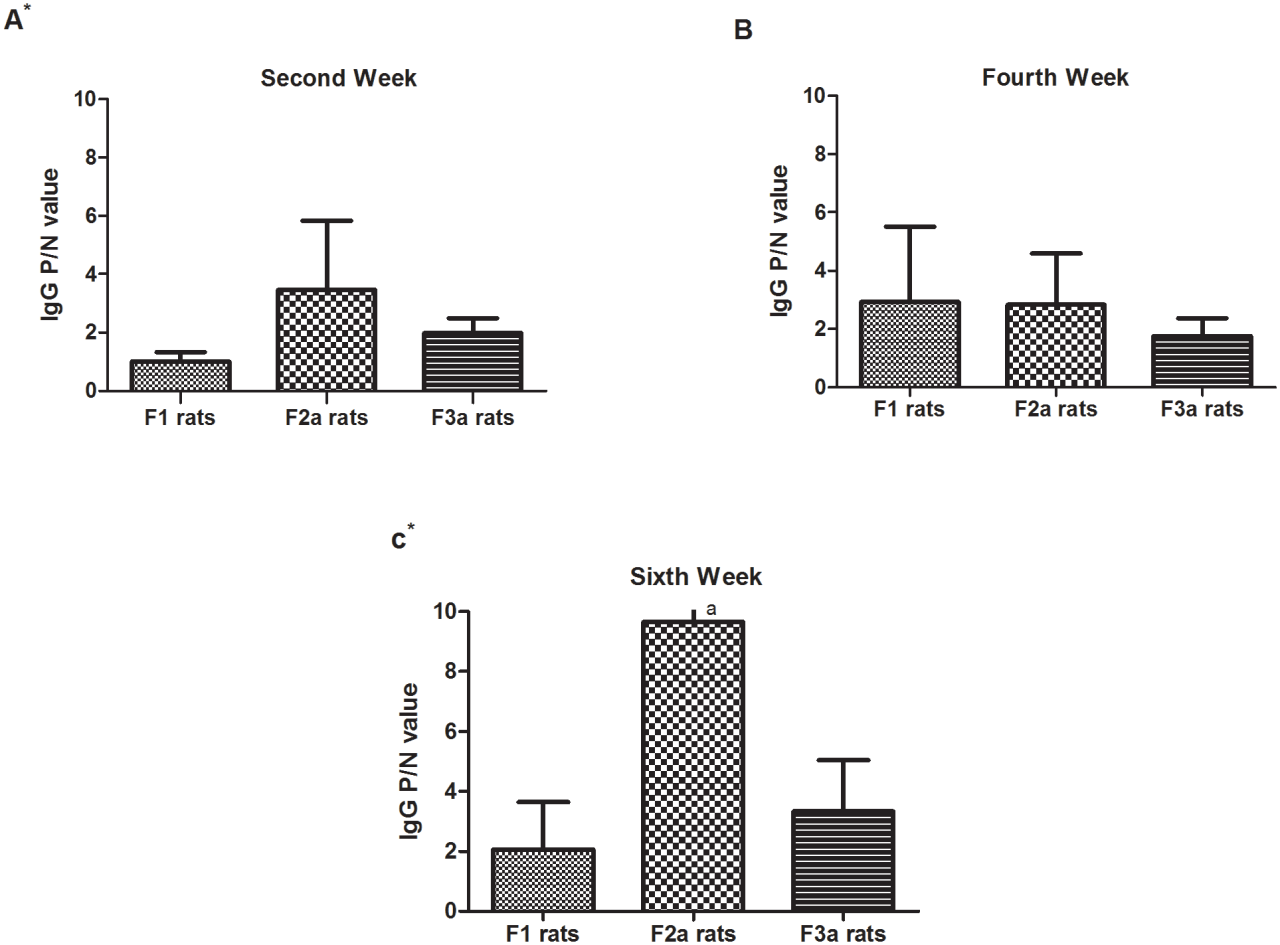
The serum IgG levels in the three generations after maternal PBS exposure. The F0 maternal OVA-exposed F1 experiment rats(F0_matOVA_), the F1 maternal PBS-exposed F2a experiment rats(F0_matOVA_/F1_matPBS_) and the F2a maternal PBS-exposedF3a experiment rats(F0_matOVA_/F1_matPBS_/F2_matPBS_) were randomly divided into OVA and non-OVA groups. The OVA and non-OVA groups were administered treatments daily for 6 weeks, and blood samples were taken on the 2^nd^(A), 4^th^(B), and 6^th^(C) weeks from the orbital plexus. The number of animals per experiment was 32, and each group contained16 animals. The serum IgG levels were measured with an ELISA technique. The data are presented as P/N values representing the ratios of the OVA group OD values divided by the non-OVA group values in the respective experiment. P/N IgG values>2 were considered to indicate positive expression levels. The statistical analysis was performed with one-way ANOVA comparisons of the three generations. *p<0.05.**(Data in** S2, S3 and S5 Tables)

As shown in Fig 6, compared with maternal OVA exposure prior to postnatal sensitization(F1), after one or two generations of maternal PBS exposure after the initial OVA exposed generation, the F2a(F0_OVAmat_/F1_PBSmat_) and F3a (F0_OVAmat_/F1_PBSmat_/F2_PBSmat_) postnatal sensitized rats showed high OVA-specific IgG levels in the sera, especially at the 2^nd^ and 6^th^ weeks. The OVA-specific IgG levels in the second-generation maternal PBS-exposed rats prior to postnatal sensitization were not higher than those in first-generation maternal PBS-exposed rats prior to postnatal sensitization. Although the IgG P/N values were not significantly different among the F1, F2a, and F3a rats in the fourth week, the positive response rates in F2a(68.8%) rats were higher than those in F3a(37.5%) and F1(43.8%) rats.

## Discussion

Previous studies have shown that food allergy in offspring is associated with maternal sensitization, especially during pregnancy and lactation. However, there is no direct evidence showing that maternal allergen exposure increases or reduces offspring immune reactions[25]. Several studies have demonstrated that maternal allergen exposure plays a harmful role in offspring responses[26], specifically as a risk factor for allergy disease development in children[27]. In contrast, other studies have shown that immune tolerance and other immunological changes are induced in offspring by long-lasting oral allergen exposure. Specifically, the preventive tolerance effects in the subsequent generation are long lasting and can be observed 4 months after birth[28]. Some research studies have attempted to reduce neonatal allergy risk through maternal allergen exposure during pregnancy and lactation[29, 30]. If the tolerance induction phenomenon is observed after maternal exposure, BN rats are not suitable for allergen models.

In our study, we used a BN rat allergen model to show that immune reactions are induced by oral OVA exposure in offspring rats after maternal oral OVA exposure during pregnancy and lactation over three generations. In particular, we found that maternal exposure to OVA significantly reduced the allergen-specific IgG levels in the offspring’s serum. This finding directly supports the assertion that maternal exposure could affect the results of immune reaction with ovalbumin protein. Unfortunately, although our results are consistent with other studies, the mechanism regarding the maternal effect on offspring is not fully understood; it is only known that food allergens and specific antibodies can cross the placenta during pregnancy[31] and be transferred by breast-feeding during lactation[32]. The fetal immune system interacts with the maternal immune system during pregnancy and lactation. In this study, we compared the sensitization of the F1, F2b, F3b rat. The OVA-specific IgG level did not significantly differ among the three sensitization groups; however, we found the lowest levels in the F3b rats, especially in the 6^th^ week. This result suggests that persistent OVA exposure in maternal rats may tend to elicit a weaker immune reaction in offspring depending on the time and frequency of stimulation.

To evaluate whether immune reaction change was maintained in the offspring, we exposed the initial maternal generation to OVA, and the first or second maternal generations were exposed to PBS for sensitization. In F2a rats, the OVA-specific IgG expression levels were high throughout the sensitization process. Previous studies have shown that allergen immune tolerance disappeared when rats are not exposed to allergen; however, the rats must be bred and raised for at least two generations on a diet free from the investigated protein under investigation[33]. In our study, OVA-specific IgG level did not significantly differ between the F2a and F3a rats, and the IgG levels in maternal rats that were not exposed for two generations were not higher than those in maternal rats that were not exposed for one generation. This result suggests that rats only need to be bred and raised for one generation to be free from allergens that could induce high IgG expression.

In conclusion, our studies demonstrate that maternal OVA exposure in BN rats affects the IgG levels of offspring rats orally sensitized with OVA. Additionally, the IgG levels of sensitized offspring rats may be influenced by maternal OVA exposure during pregnancy and lactation. Moreover, after breeding the rats in non-allergen conditions for one generation, a positive IgG response is induced in offspring rats that are orally sensitized.

This study only studied the changes in IgG levels between different generations. However, the IgE levels, anaphylactic symptoms are important parameters for evaluating allergen sensitization in animal model; In our results, the anaphylactic reaction is not strong, the IgE parameter and anaphylactic symptoms were not measured. In the future, we will use a high-sensitivity method to detect mild anaphylactic reactions and evaluate the changes in allergy-relevant parameters in different generations.

## Supporting Information

S1 File Editorial certificate: the manuscript is edited by American Journal Experts. (PDF)

S1 Table The serum IgG levels in first-generation control experiment rats. (DOC)

S2 Table The serum IgG levels in first-generation F1 experiment rats. (DOC)

S3 Table The serum IgG levels in second-generation F2a experiment rats. (DOC)

S4 Table The serum IgG levels in second-generation F2b experiment rats. (DOC)

S5 Table The serum IgG levels in third-generation F3a experiment rats. (DOC)

S6 Table The serum IgG levels in third-generation F3b experiment rats. (DOC)
