# Supplementary material for: IgG Expression upon Oral Sensitization in Association with Maternal Exposure to Ovalbumin
Source: PLoS One. 2016 Feb 4;11(2):e0148251. doi: 10.1371/journal.pone.0148251 (PMC4742080; doi:10.1371/journal.pone.0148251)
Supplement: S1 Table — (DOC) [file pone.0148251.s002.doc]

S1 Table. The serum IgG levels in first-generation control experiment rats

| case | P/N value | | |
| --- | --- | --- | --- |
|  | Second Week | Fourth Week | Sixth Week |
| 1 | 2.29 | 2.13 | 1.87 |
| 2 | 0.71 | 4.84 | 2.83 |
| 3 | 1.14 | 1.19 | 1.56 |
| 4 | 0.73 | 4.59 | 2.00 |
| 5 | 1.22 | 3.20 | 1.91 |
| 6 | 1.60 | 5.06 | 2.49 |
| 7 | 2.51 | 6.96 | 2.34 |
| 8 | 2.31 | 5.35 | 2.69 |
| 9 | 0.88 | 4.84 | 3.09 |
| 10 | 0.55 | 2.92 | 2.28 |
| 11 | 1.50 | 4.70 | 2.31 |
| 12 | 2.38 | 4.86 | 2.25 |
| 13 | 0.96 | 4.60 | 2.58 |
| 14 | 4.10 | 4.73 | 2.24 |
| 15 | 4.00 | 6.39 | 2.54 |
| 16 | 3.72 | 5.26 | 2.89 |
