# Supplementary material for: IgG Expression upon Oral Sensitization in Association with Maternal Exposure to Ovalbumin
Source: PLoS One. 2016 Feb 4;11(2):e0148251. doi: 10.1371/journal.pone.0148251 (PMC4742080; doi:10.1371/journal.pone.0148251)
Supplement: S2 Table — (DOC) [file pone.0148251.s003.doc]

S2 Table. The serum IgG levels in first-generation F1 experiment rats

| case | P/N value | | |
| --- | --- | --- | --- |
|  | Second Week | Fourth Week | Sixth Week |
| 1 | 1.62 | 4.16 | 2.40 |
| 2 | 0.80 | 1.17 | 1.04 |
| 3 | 0.73 | 1.43 | 0.84 |
| 4 | 0.76 | 1.02 | 0.73 |
| 5 | 0.69 | 1.11 | 0.83 |
| 6 | 1.41 | 7.31 | 4.21 |
| 7 | 0.55 | 1.04 | 0.83 |
| 8 | 1.26 | 2.77 | 1.09 |
| 9 | 1.09 | 7.93 | 4.67 |
| 10 | 1.18 | 5.43 | 4.00 |
| 11 | 0.83 | 0.83 | 0.84 |
| 12 | 0.80 | 0.36 | 0.57 |
| 13 | 1.05 | 3.53 | 3.64 |
| 14 | 0.98 | 0.76 | 1.58 |
| 15 | 1.42 | 6.67 | 4.66 |
| 16 | 1.09 | 1.22 | 1.01 |
