# Supplementary material for: IgG Expression upon Oral Sensitization in Association with Maternal Exposure to Ovalbumin
Source: PLoS One. 2016 Feb 4;11(2):e0148251. doi: 10.1371/journal.pone.0148251 (PMC4742080; doi:10.1371/journal.pone.0148251)
Supplement: S3 Table — (DOC) [file pone.0148251.s004.doc]

S3 table. The serum IgG levels in second-generation F2a experiment rats

| case | P/N value | | |
| --- | --- | --- | --- |
|  | Second Week | Fourth Week | Sixth Week |
| 1 | 3.83 | 2.56 | 1.57 |
| 2 | 2.82 | 5.11 | 19.00 |
| 3 | 5.87 | 4.11 | 11.63 |
| 4 | 7.41 | 4.96 | 16.42 |
| 5 | 6.79 | 5.44 | 15.91 |
| 6 | 6.59 | 5.22 | 15.24 |
| 7 | 4.24 | 2.57 | 12.73 |
| 8 | 0.84 | 0.97 | 0.92 |
| 9 | 1.03 | 1.35 | 4.07 |
| 10 | 0.89 | 0.67 | 0.99 |
| 11 | 1.31 | 1.80 | 11.58 |
| 12 | 5.16 | 2.83 | 10.04 |
| 13 | 0.95 | 0.56 | 0.90 |
| 14 | 0.82 | 0.64 | 3.58 |
| 15 | 2.80 | 3.49 | 14.62 |
| 16 | 4.00 | 3.12 | 15.34 |
