# Supplementary material for: IgG Expression upon Oral Sensitization in Association with Maternal Exposure to Ovalbumin
Source: PLoS One. 2016 Feb 4;11(2):e0148251. doi: 10.1371/journal.pone.0148251 (PMC4742080; doi:10.1371/journal.pone.0148251)
Supplement: S4 Table — (DOC) [file pone.0148251.s005.doc]

S4 table. The serum IgG levels in second-generation F2b experiment rats

| case | P/N value | | |
| --- | --- | --- | --- |
|  | Second Week | Fourth Week | Sixth Week |
| 1 | 0.49 | 0.40 | 3.02 |
| 2 | 1.83 | 4.76 | 5.13 |
| 3 | 0.42 | 3.99 | 4.55 |
| 4 | 0.09 | 4.25 | 3.01 |
| 5 | 0.00 | 0.56 | 2.44 |
| 6 | 0.86 | 3.14 | 4.03 |
| 7 | 1.37 | 4.72 | 4.97 |
| 8 | 0.18 | 0.88 | 3.71 |
| 9 | 3.99 | 4.94 | 5.48 |
| 10 | 4.22 | 4.60 | 4.70 |
| 11 | 0.55 | 0.65 | 0.80 |
| 12 | 0.33 | 5.14 | 4.51 |
| 13 | 3.94 | 5.33 | 4.60 |
| 14 | 0.89 | 4.47 | 3.88 |
| 15 | 1.46 | 1.11 | 1.72 |
| 16 | 3.94 | 2.65 | 3.41 |
