# Supplementary material for: IgG Expression upon Oral Sensitization in Association with Maternal Exposure to Ovalbumin
Source: PLoS One. 2016 Feb 4;11(2):e0148251. doi: 10.1371/journal.pone.0148251 (PMC4742080; doi:10.1371/journal.pone.0148251)
Supplement: S5 Table — (DOC) [file pone.0148251.s006.doc]

S5 table. The serum IgG levels in third-generation F3a experiment rats

| case | P/N value | | |
| --- | --- | --- | --- |
|  | Second Week | Fourth Week | Sixth Week |
| 1 | 2.59 | 1.89 | 4.69 |
| 2 | 2.27 | 1.89 | 4.45 |
| 3 | 1.86 | 2.78 | 4.74 |
| 4 | 2.46 | 2.57 | 4.99 |
| 5 | 2.06 | 1.46 | 5.11 |
| 6 | 1.68 | 1.42 | 1.46 |
| 7 | 1.84 | 2.31 | 3.88 |
| 8 | 1.29 | 1.14 | 0.93 |
| 9 | 2.57 | 1.84 | 4.36 |
| 10 | 1.18 | 0.96 | 3.20 |
| 11 | 2.07 | 2.08 | 4.25 |
| 12 | 0.97 | 2.00 | 4.16 |
| 13 | 2.31 | 0.83 | 1.05 |
| 14 | 2.21 | 0.92 | 0.72 |
| 15 | 2.45 | 1.43 | 0.72 |
| 16 | 2.05 | 2.51 | 4.59 |
