# Supplementary material for: IgG Expression upon Oral Sensitization in Association with Maternal Exposure to Ovalbumin
Source: PLoS One. 2016 Feb 4;11(2):e0148251. doi: 10.1371/journal.pone.0148251 (PMC4742080; doi:10.1371/journal.pone.0148251)
Supplement: S6 Table — (DOC) [file pone.0148251.s007.doc]

S6 table. The serum IgG levels in third-generation F3b experiment rats

| case | P/N value | | |
| --- | --- | --- | --- |
|  | Second Week | Fourth Week | Sixth Week |
| 1 | 2.22 | 5.00 | 2.08 |
| 2 | 1.74 | 4.37 | 1.75 |
| 3 | 1.93 | 2.84 | 1.13 |
| 4 | 0.95 | 4.91 | 1.46 |
| 5 | 0.39 | 0.48 | 0.32 |
| 6 | 1.73 | 4.49 | 1.73 |
| 7 | 1.38 | 0.94 | 0.41 |
| 8 | 0.78 | 0.64 | 2.53 |
| 9 | 1.59 | 1.74 | 1.23 |
| 10 | 2.63 | 4.84 | 1.58 |
| 11 | 0.84 | 0.83 | 0.56 |
| 12 | 0.37 | 0.39 | 0.75 |
| 13 | 0.36 | 0.41 | 0.72 |
| 14 | 0.31 | 0.31 | 1.04 |
| 15 | 1.44 | 1.12 | 0.83 |
| 16 | 0.43 | 0.52 | 0.96 |
